# The Effect of Sample Pretreatment on the Anthocyanin Content in Czech Wild Elderberry (*Sambucus nigra* L.)

**DOI:** 10.3390/molecules28186690

**Published:** 2023-09-19

**Authors:** Lenka Česlová, Petra Kalendová, Lucie Dubnová, Marek Pernica, Jan Fischer

**Affiliations:** 1 Department of Analytical Chemistry, Faculty of Chemical Technology, University of Pardubice, Studentská 573, 53210 Pardubice, Czech Republic; luci-dubi@seznam.cz (L.D.); jan.fischer@upce.cz (J.F.); 2 Department of Inorganic Technology, Faculty of Chemical Technology, University of Pardubice, Doubravice 41, 53210 Pardubice, Czech Republic; petra.kalendova@upce.cz; 3 Research Institute of Brewing and Malting, Mostecká 7, 61400 Brno, Czech Republic; pernica@beerresearch.cz

**Keywords:** elderberry (*Sambucus nigra* L.), anthocyanins, HPLC, total anthocyanin content, total phenolic content, antioxidant capacity, principal component analysis

## Abstract

This work focusses on the analysis of anthocyanins present in wild Czech elderberries, using spectrophotometric methods and liquid chromatography. Spectrophotometric methods were used to determine antioxidant capacity, total phenolic content, and total anthocyanin content. Further, four major elderberry anthocyanins were determined using reversed-phase high-performance liquid chromatography with isocratic elution of 30% aqueous methanol with 5% of formic acid. All optimised methods were applied to the analysis of extracts prepared from frozen and dried elderberry fruit samples, and the results were evaluated using principal component analysis which clearly divided the samples into individual groups according to the sample pretreatment (frozen and dried samples). The frozen samples reached higher values of antioxidant capacity and total phenolic and anthocyanin contents compared to the dried samples, probably due to the degradation of anthocyanins during the drying process.

## 1. Introduction

The European elderberry (*Sambucus nigra* L.) is a deciduous tree-like shrub, widespread on almost every continent of the world. Creamy white flowers bloom from May to July and produce purple-black berries from August to late September. The berries grow in clusters and have been used for centuries for medicinal purposes and are traditionally consumed to prevent or diminish the symptoms of several diseases [1,2,3,4]. Elderberries contain many bioactive compounds, mainly anthocyanin derivatives, including cyanidin 3-glucoside, cyanidin 3-sambubioside, cyanidin 3-rutinoside, cyanidin 3-sambubioside-5-glucoside, and cyanidin 3,5-diglucoside, with cyanidin 3-sambubioside or cyanidin 3-glucoside being the most abundant depending on the variety and ripeness [4,5,6,7,8]. 

At present, anthocyanins are becoming very popular because they are potential natural alternatives to synthetic food colourants, and, in addition, their antioxidant capacity plays an important role in the prevention of different diseases [4,9]. Due to the high anthocyanin content, elderberry may be an excellent source of these natural colourants in the food industry [6,10]. However, anthocyanins are very unstable and degrade easily. Their stability is mainly influenced by the pH value of the given system, and therefore their use as food colourants is considerably limited. The pH value of the system affects not only their structure but also their colour, and depending on pH, they occur in four different structures with a characteristic colour. Anthocyanins are most stable at low pH values (pH < 3), where they occur as the red flavylium cation [4,11]. Due to their high stability under acidic conditions, anthocyanins can be successfully used to colour foods with a low pH level, such as soft drinks, conserves, water ice creams, table jellies, sauces, and confectionery [12,13,14]. In addition to pH, their stability is also affected by temperature, oxygen, light, enzymes, and other accompanying substances such as ascorbic acid, sugars, sulphites, copigments, and metal ions, i.e., matrix compounds [9,11,15]. Furthermore, the water activity of the systems and the concentration of anthocyanins affect their colour stability [16]. 

One of the most important quality attributes of anthocyanin food products is their stability during processing and storage; therefore, some studies have reported the effect of processing and storage on the anthocyanin content in fruit juice [9,12,17,18,19,20,21]. However, the works focused on the change in anthocyanin content during pretreatment of elderberry fruits are limited [22]. Therefore, the objective of this work is to monitor the effect of different sample pretreatment methods before the storage of elderberry fruits (drying vs. freezing) on anthocyanin content, using rapid and simple chromatographic and spectrophotometric methods.

## 2. Results and Discussion

### 2.1. Determination of Antioxidant Capacity

**Figure 1:**
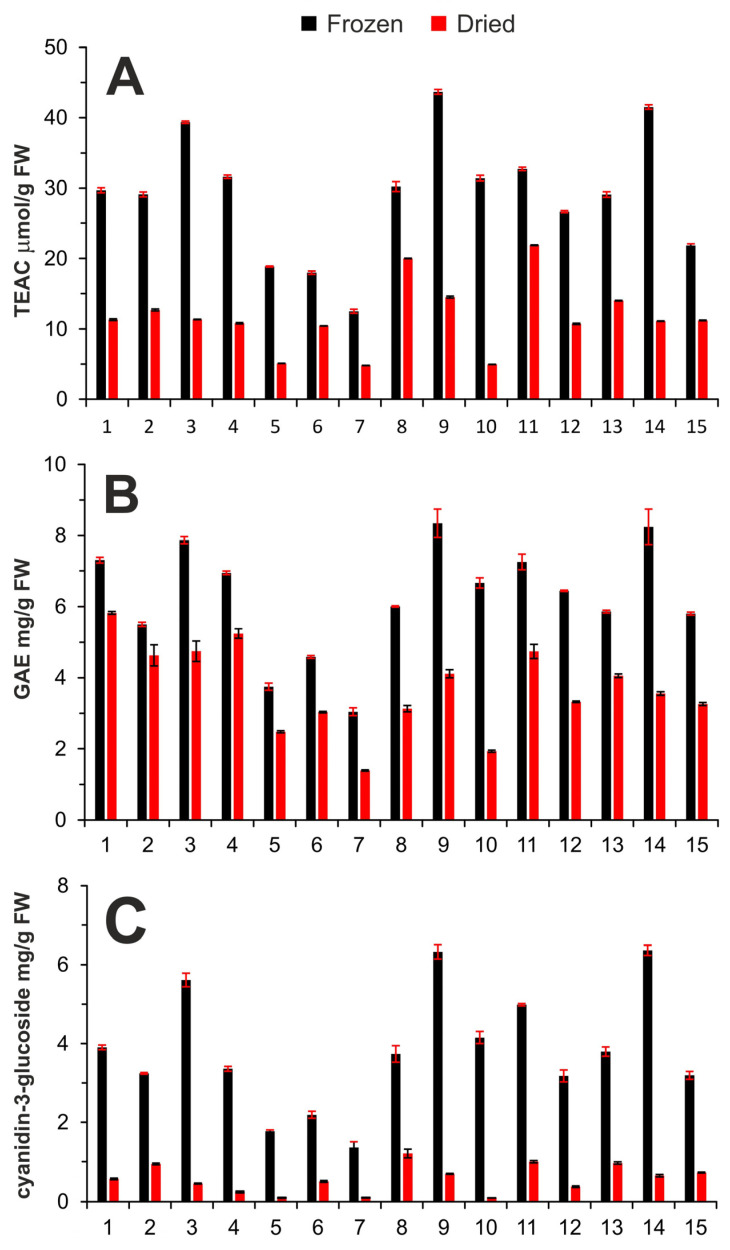
Antioxidant capacity (**A**), total phenolic content (**B**), and total anthocyanin content (**C**) of individual elderberry extracts.

Antioxidant capacity of the sample extracts (Table 1) were measured according to the ABTS method described in Materials and Methods Section (Section 3.4), and the results are expressed as Trolox equivalent antioxidant capacity (TEAC) given as μmol of Trolox in 1 g of fresh sample (FW). Significant differences were observed between the TEAC values of individual samples and especially between the frozen and dried samples (Figure 1A). The TEAC values of frozen samples were in the range of 12.5–43.7 μmol/g FW, while lower TEAC values were observed for dried samples, ranging from 6.6 to 29.0 μmol/g FW. After drying, the antioxidant capacity decreases by approximately 60% (Figure 1A), probably due to the degradation of compounds with antioxidant properties. Similar TEAC values, 3.2–39.6 μmol/g FW, were determined in frozen fruits of different elderberry species or interspecific hybrids [23]. However, more than two times higher TEAC values, 109 μmol/g FW, were presented in the study by Silva et al. [24], who used freeze-dried material for extraction. Freeze-drying is probably gentler and protects the antioxidants present in the treated material, and therefore the antioxidant capacity reaches higher values. The difference in antioxidant capacity can be further caused by different varieties and growing locations of elderberries [23,24,25]. Moreover, the difference in the antioxidant capacity of wild and orchard-grown elderberries, with orchard elderberries providing higher TEAC values, was observed in the study of Młynarczyk et al. [25].

### 2.2. Determination of the Total Phenolic Content

The total phenolic content (TPC) was measured in all extracts, using the method described in Materials and Methods Section (Section 3.4), and the results are expressed as gallic acid equivalent (GAE) given as mg of gallic acid in 1 g of fresh sample. GAE values varied significantly between individual samples, which is evident from Figure 1B. This difference could be caused by the harvesting time and location altitude, which affect the ripening of the berry and thus the content of phenolic compounds [6]. Moreover, the difference between the frozen and dried samples was again observed (Figure 1B). The mean GAE values of the frozen and dried samples extracts were in the ranges of 3.0–8.4 mg/g FW and 1.4–5.8 mg/g FW, respectively. Therefore, freezing can be considered as a better storage condition compared to drying. 

Similar total polyphenol values were recorded for elderberries in the other studies [6,8,23,26] ranging from 3.5 to 7.0 mg/g of fresh sample (FW). The higher GAE values about 11 mg/g were presented by Silva et al. [24], who used a freeze-dried material for extraction same as Wu et al. who presented even 19.4 mg/g of GAE [7]. A significant difference was observed between the GAE values of various cultivars in the study by Lee et al. [6], where the influence of the harvest year and the ripening was also evident. The effect of the harvest year and thus climatic conditions on the content of phenolic compounds was further apparent in the study by Fereira et al. [27]. The cultivated elderberry plant usually contains a higher amount of phenolic compounds than the wild plant [25], which is apparent from the studies of Fereira et al. [27] and Csorba et al. [28], who examined different cultivars of elderberry fruits, and the GAE values ranged 8.2–14.8 mg/g FW and 8.5–20.2 mg/g FW, respectively.

### 2.3. Determination of Total Anthocyanin Content

The pH-differential method [29] with two buffers differing in pH values (pH 1 and pH 4.5) was used for total anthocyanin content (TAC) assessment. The optimal volume of the sample extract added to the buffers was determined by its gradually increasing amount and monitoring the change in absorbance. According to the literature [4,5,6,7,8,10,24,26] and our finding, cyanidin-3-glucoside and cyanidin-3-sambubioside are the main anthocyanins present in elderberries. For this reason, cyanidin-3-glucoside (C-Glu) is frequently used as a standard for TAC determination [6,24,25,26,27,28]. The mean TAC values of all the extracts measured are shown in Figure 1C. The TAC values strongly differ between the measured samples, and the results are consistent with the antioxidant capacity and TPC because anthocyanins are the main phenolic compounds present in elderberries with high antioxidant activity. Very interesting finding is the extremely high decrease in TAC after drying. The TAC decreased from values between 1.4 and 6.4 mg of C-Glu/g FW in the frozen samples to 0.1 and 1.0 mg of C-Glu/g in the dried samples, which is an up to 90% decrease. The drying process, even very gentle, has a strong negative impact on the anthocyanin content, which consequently influences the antioxidant capacity and TPC of the berries (Figure 1). 

The TAC values of the frozen samples agree well with the already published results [6,8,24,25,26] that are usually between 1.1 and 8.1 mg of C-Glu/g FW. In the study by Mlynarczyk et al. [25], the TAC values are given as dry matter of the sample. However, when we assume a water content of 80%, the reported value of 30.7 mg/g dry matter is approximately 6.1 mg/g of fresh sample, which is consistent with our results. Higher TAC values (4.4 to 14.1 mg of C-Glu/g FW) were reported in cultivated elderberry plants [27,28] with considerable differences between the cultivars. Similar to the polyphenol content, the TAC values are influenced by the harvest year [6,27].

### 2.4. Qualitative and Quantitative HPLC Analysis of Anthocyanins

#### 2.4.1. Optimization of HPLC Separation

**Figure 2:**
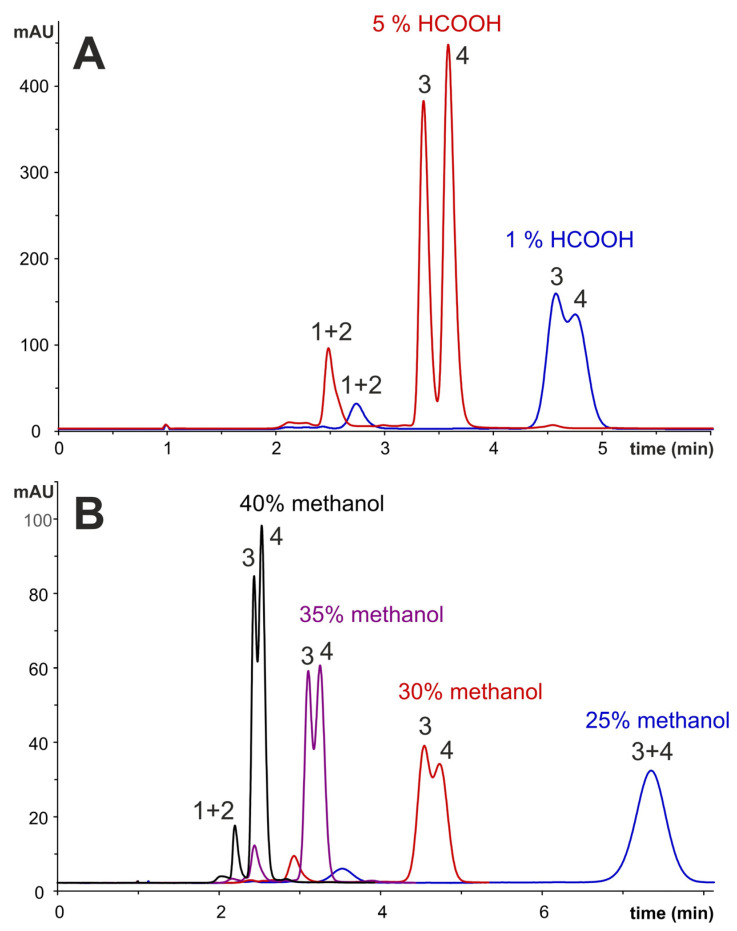
Chromatographic separation of main anthocyanins present in elderberry extract of sample 1F. (**A**) Effect of acidification of mobile phase on anthocyanin separation, mobile phase: 30% methanol acidified by 5% HCOOH (red) and 1% HCOOH (blue); (**B**) the influence of methanol concentration in mobile phase containing 1% of HCOOH on separation of anthocyanins (blue—25%, red—30%, purple—35%, and black—40% methanol). Column: Ascentis Express C18 (150 × 3.0 mm, 3 μm), T = 30 °C, F = 0.4 mL/min, 520 nm; (1) cyanidine-3-sambubioside-5-glucoside, (2) cyanidine-3,5-diglucoside, (3) cyanidine-3-sambubioside, (4) cyanidine-3-glucoside.

The aim of the optimisation was the separation of the present anthocyanins in a short time, using an isocratic elution with the best resolution. Aqueous methanol was chosen as the mobile phase, and the Ascentis Express column with octadecyl silica gel as a stationary phase was used. The influence of the concentration of methanol and the acidification of the mobile phase on the separation was tested within optimisation and is shown in Figure 2. 

Due to the higher stability of anthocyanins as flavylium cations under acidic conditions (Figure S1) [4,11], the mobile phase was acidified using 1% or 5% (*v*/*v*) of formic acid. A higher resolution of individual peaks was achieved with a higher formic acid content in the mobile phase (Figure 2A). Furthermore, with increasing acidification, absorbance increases (Figure S1), which affects the sensitivity of the determination. In other studies, even 10% of formic acid was added to the mobile phase [6,26,30]; however, in those cases, special columns with high stability at lower pH had to be used because conventional reversed-phase columns are stable at pH = 2–8, and at pH < 2 hydrolysis of silica gel occurs, leading to a prolonged retention times and reduced columns lifetime. The influence of methanol concentration in the mobile phase is illustrated in Figure 2B. With an increasing concentration of methanol, the resolution increases, and the retention time decreases. The best separation conditions were achieved with 30% methanol acidified by 5% of formic acid, where the unit resolution of the main two anthocyanins was achieved. The gradient elution was also tested to improve the resolution of the first eluted minor anthocyanins. However, the separation did not improve significantly; therefore, isocratic elution, where long equilibration of the column is not necessary, was finally selected for the analysis. The separation took less than 5 min, and the resolution of separated anthocyanins was the same or even better contrary to the works already present, where a long gradient elution was applied for 25–70 min [5,6,7,26,30,31]. The baseline separation of the four main anthocyanins was achieved only in the work of Silva et al. [24], but the 70 min separation was necessary.

#### 2.4.2. Identification and Quantification of Anthocyanins

Four main anthocyanins, two major ones being cyanidin-3-sambubioside and cyanidin-3-glucoside and two minor ones being cyanidine-3-sambubioside-5-glucoside and cyanidine-3,5-diglucoside, are present in the fruits of *Sambucus nigra*. The identification of individual anthocyanins was based on the comparison of retention times with standards and according to the literature [1,6,7,8,24,26,30,31,32]. The trace level of the other three anthocyanins (cyanidin-3-rutinoside, pelargonidin-3-glucoside, and pelargonidin-3-sambubioside) were found in other studies [7,32]. Unlike European elderberry cultivars (*S. nigra*), cyanidine-3-sambubioside-5-glucoside and especially cyanidin-3-(E)-*p*-coumaroyl-sambubioside-5-glucoside are the main anthocyanins in American elderberry cultivars (*S. canadensis*) [6]. In total, 19 anthocyanins were identified and quantified in four *Sambucus* species (*S. cerulea*, *S. ebulus*, *S. nigra*, and *S. racemosa*) and eight hybrids, using HPLC/MS [32]. Cyanidin-3-(E)-*p*-coumaroyl-sambubioside-5-glucoside was the predominant anthocyanin in hybrids of *S. javanica* [32] as in American cultivars (*S. canadensis*) [6]. 

**Figure 3:**
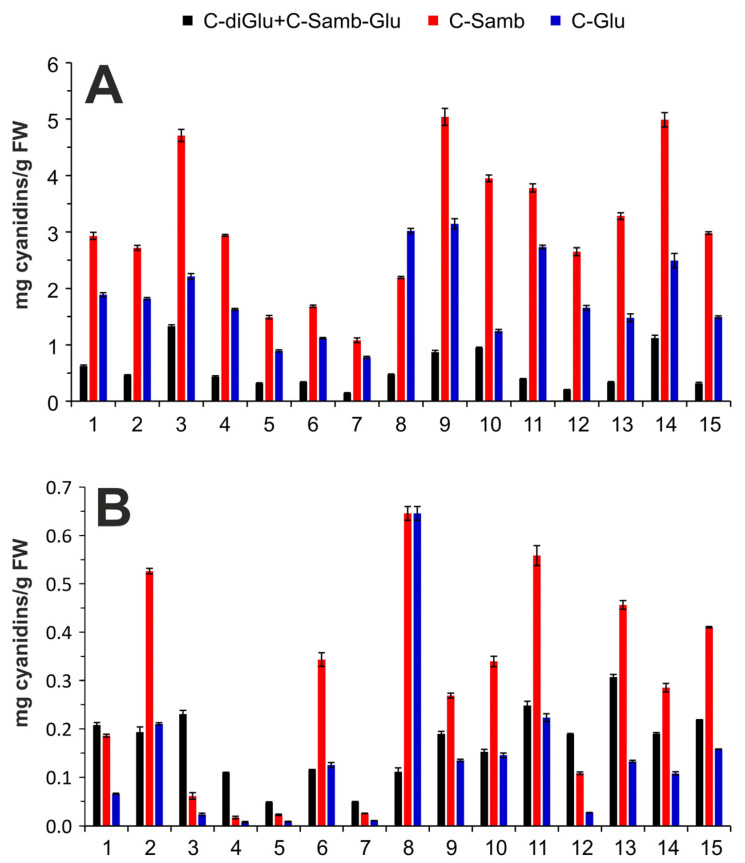
Anthocyanin contents in frozen (**A**) and dried (**B**) samples. Cyanidine-3-sambubioside-5-glucoside (C-Samb-Glu); cyanidine-3,5-diglucoside (C-diGlu); cyanidine-3-sambubioside (C-Samb); cyanidine-3-glucoside (C-Glu).

The quantitative analysis of the main four anthocyanins was performed using the external standard calibration method using commonly available standards. Cyanidine-3,5-diglucoside was used for quantification of cyanidine-3-sambubioside-5-glucoside, and both derivatives were quantified together, because their separation was not complete. The individual calibration parameters, together with their standard deviations, coefficient determination, and detection and quantification limits, are shown in Table S1. The natural plant water content plays an important role in the calculation and comparison of analyte concentrations in the dried and frozen samples. Therefore, the water content was determined in individual samples, and the exact values of the dry matter of individual samples are shown in Table 1. The dry matter was subsequently used for the conversion of the results of the dried samples to fresh weight. The quantitative results of all measured samples related to a gramme of fresh material are depicted in Figure 3. 

The main anthocyanin was cyanidin-3-sambubioside followed by cyanidin-3-glucoside in almost all samples studied, which is consistent with the results of other authors [1,8,24,26,30,31]. The exception was sample No. 8, where cyanidin-3-glucoside was the dominant anthocyanin, as in the works of Lee et al. [6] and Wu et al. [7]. 

The content of anthocyanins in elderberry can be influenced by several factors such as cultivar, ripeness, harvest year, and climatic conditions [25,27,28]. Moreover, the berries have a higher content of anthocyanins and other polyphenols when grown in a well-organized orchard compared to those in the wild berries [22,25]. Therefore, a very different amount of monitored anthocyanins were determined in individual samples (Figure 3). The content of cyanidin-3-sambubioside and cyanidin-3-glucoside in frozen samples ranged from 1.1 to 5.04 mg/g FW and from 0.8 to 3.2 mg/g FW, respectively, and represents even 81.5% (*w*/*w*) of all anthocyanins quantified in the present study (Figure 3A). The two minor anthocyanins, cyanidin-3,5-diglucoside and cyanidine-3-sambubioside-5-glucoside, together occupy less than 20% (*w*/*w*) of the sample, and their content was in the range of 0.14–1.33 mg/g FW.

During drying, very high decrease in anthocyanin content was observed, and their overall content in dried samples did not exceed 1.4 mg/g FW (Figure 3B). The concentration decrease in two major cyanidin derivatives was more significant than that in the minor derivatives; therefore, their percentage content in the dried samples was 54% (*w*/*w*) which is similar to that of the minor cyanidin derivatives (Figure 3B). The drying process can probably cause some degradation or transformation of anthocyanins [1], even under gentle conditions.

#### 2.4.3. Data Evaluation Using Statistical Methods

First, the variability and normality of the data obtained using spectrophotometric and chromatographic methods (ABTS, TPC, TAC, and HPLC) were assessed using descriptive statistics (Kolmogorov–Smirnov test and graphical methods such as P-P graph, Q-Q graph, and histograms). Due to the high differences in the concentration levels of individual methods, it was necessary to standardise the data. 

**Figure 4:**
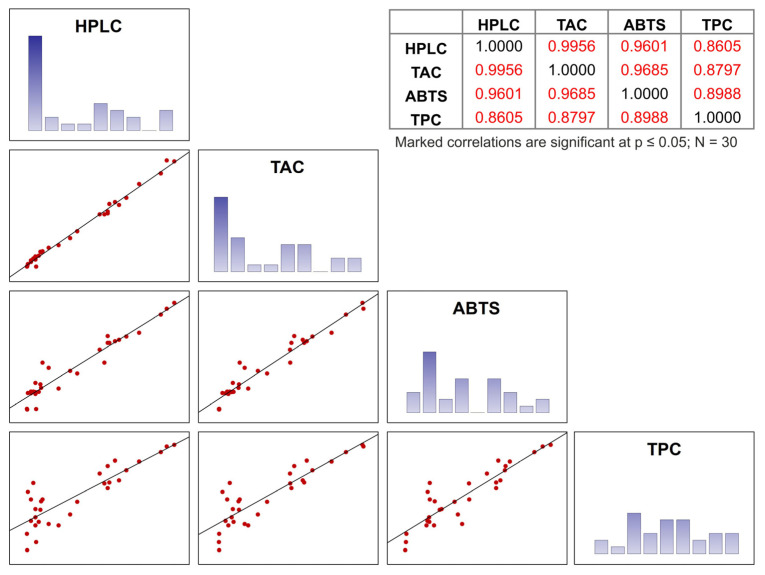
The scatterplot of the correlation matrix and correlation table of spectrophotometric methods and HPLC analysis.

The results obtained using different spectrophotometric methods (TPC, TAC, and ABTS) and the total amount of anthocyanins determined by HPLC are significantly correlated (Figure 4) and therefore very similar to each other. High correlation was observed especially between the TAC and HPLC results, where the correlation coefficient was higher than 0.99. The spectrophotometric pH differential method can therefore be used for rapid screening of anthocyanin content with accurate results. The results of the antioxidant capacity measured by the ABTS method correlate with the HPLC and the TAC method with a correlation coefficient higher than 0.96. These considerable correlations are probably caused by the high scavenging capacity of anthocyanins against the ABTS radical. The lowest correlation coefficients below 0.9 were recorded between the results obtained by the TPC methods and the rest of the methods applied because this method can also be sensitive to other compounds present in the samples, such as ascorbic or other organic acids [33].

**Figure 5:**
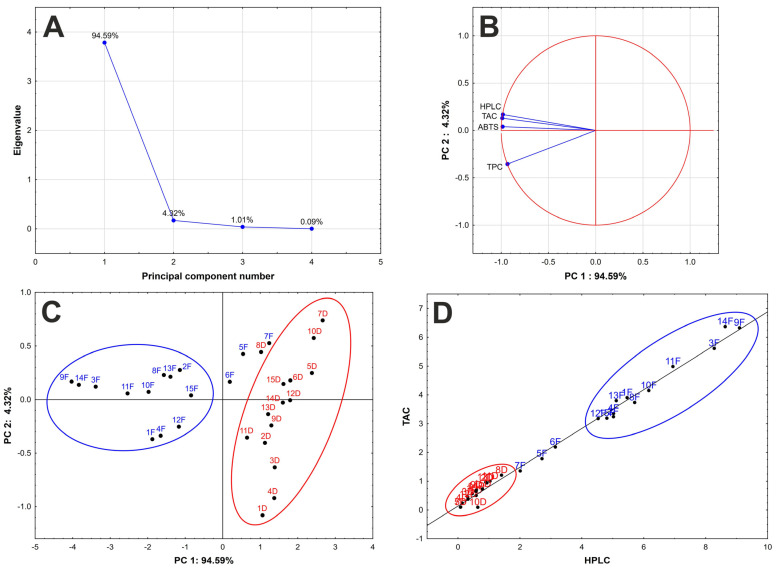
The statistical data treatment: (**A**) Cattel graph, (**B**) principal component loading, (**C**) principal component score, (**D**) plot of correlation between HPLC and TAC (F = frozen, D = dried).

Further, the data obtained using all methods were statistically evaluated and compared by a principal component analysis. This multivariate statistical method was successfully applied to distinguish elderberry samples according to harvest year [27]. The importance of individual principal components (PC) was distinguished from the scree plot (Figure 5A). The first principal component covers 94% of total data variance, and together with PC2, almost 99% of the system could be described. From the principal component loading (Figure 5B), the high importance of all variables for PC1 description is evident (long vector); however, PC2 is given mainly by the TPC results. Further, the above-mentioned correlation of the TAC, ABTS, and HPLC results was confirmed by principal component loading (Figure 5B), where the vectors are very close to each other, indicating great similarity in the data obtained. 

From the scatterplots of the principal component score (Figure 5C), it is evident that the dried and frozen samples can be divided into discrete groups according to the content of anthocyanins in PC1 direction and the content of other phenolic compounds, which can be determined by TPC method, in PC2 direction. In general, the samples with high content of anthocyanins are located on the left side of the plot, and samples with high GAE values at the bottom of the plot. Naturally, the frozen samples (F) are located to the left of the scatterplot, and the dried samples (D) are located to the right. Moreover, due to the low content of anthocyanins in dried samples, these are divided according to GAE values in PC2 direction. The frozen samples 5F, 6F, and 7F are located near dried samples (Figure 5C) with a low content of anthocyanins and can be considered as outliers in the group of frozen samples. These samples were grown at the highest altitude (Table 1), so the ripeness of the berries was probably lower at the same harvest time, and therefore the anthocyanin content of these samples is the lowest of all samples studied (Figure 1). Further, sample 15F was also grown at a higher altitude and is therefore located close to the samples 5F, 6F, and 7F (Figure 5C). Accordingly, we assume that the content of anthocyanins is strongly influenced by the ripeness of the berries and consequently by the altitude at which they grow. The effect of other geographical parameters (latitude and longitude) on the content of anthocyanins, and thus the distribution of the samples, was not proven. 

In the case of dried samples, the sample 8D is slightly distant from the rest of dried samples (Figure 5C) due to the lower degradation of anthocyanins observed after drying of the berries (Figure 1). 

Similar discrimination of the frozen and dried samples was achieved using common correlation of the TAC and HPLC results (Figure 5D), where the samples are located along the line according to the content of anthocyanins. The dried samples are located at the bottom of the plot, and the frozen samples at the top. Samples 5F,6F, and 7F are in the middle and can be considered as outliers. 

Moreover, the analysis of variance was applied to verify the significance of the sample pretreatment on the content of anthocyanins. From the ANOVA results shown in Figure S2 and Table S2, it is evident that the sample pretreatment has a significant effect on the result obtained.

## 3. Materials and Methods

### 3.1. Chemicals

Standards of anthocyanins (cyanidin-3-sambubioside, cyanidin-3,5-diglucoside, cyanidin-3-glucoside) were purchased in Extrasynthese (Genay, France). Trolox, gallic acid, ABTS, 2 M Folin–Ciocalteu reagent (all Sigma Aldrich, St. Louis, MO, USA), potassium persulfate (Laborchemie, Apolda, Germany), potassium chloride, sodium acetate, and sodium carbonate (all Lach-ner, Neratovice, Czech Republic) were used for spectrophotometric measurements. Methanol, formic acid (both Sigma Aldrich), and deionized water (Milli-Q purification system, Merck Millipore, Darmstadt, Germany) were used for extraction and separation. 

### 3.2. Sample Preparation

The fruits of wild elderberry (Table 1) were collected in different places in the Czech Republic during one week in September. First, the samples were weighted, and one part of the samples was dried in an oven at 30 °C, and the other part was frozen at −18 °C. The frozen samples were kept in a freezer for three months, while the dried samples were stored in a container in a dry and dark place at room temperature before the analysis. In the case of dried samples, the drying matter calculated after drying (Table 1) was used for conversion of the results to the fresh weight. 

After three months of storage, 1 g of dried or frozen samples were ground and then mixed with 10 mL of aqueous methanol (70%, *v*/*v*) acidified by formic acid (5%, *v*/*v*). The extraction was carried out in an ultrasonic bath three times for 15 min, and supernatants were combined. Before analysis, extracts were centrifuged for 10 min at 5000 rpm and filtrated through a 0.45 μm nylon filters and diluted as necessary by a formic acid water solution (5%, *v*/*v*). 

### 3.3. HPLC Analysis

The HPLC system was equipped with an LC-20AD binary gradient pump, a DGU-20A5 degassing unit, a spectrophotometric detector SPD-20A (all Shimadzu, Kyoto, Japan), a 7725i manual injection valve with 5 μL injection loop (Rheodyne, Rohnert Park, CA, USA), and an LCO 102 column thermostat (Ecom, Prague, Czech Republic). Separation of anthocyanins was performed on column Ascentis Express C18 (150 mm × 3 mm i.d., 3.0 μm particle size; Supelco, St. Louis, MO, USA) with isocratic elution of the mobile phase consisting of 30:70 (*v*/*v*) methanol:water. Both solvents of the mobile phase were acidified with formic acid (5%, *v*/*v*). The flow rate was 0.5 mL/min, the temperature was 30 °C, injection volume 5 μL, and detection wavelength 520 nm. 

### 3.4. Spectrophotometric Analysis

The determination of antioxidant capacity, total anthocyanin content, and total phenolic content was carried out with a UV-2450 spectrophotometer (Shimadzu), using a 1 cm S/G10 glass cuvette from Fisher Scientific (Pardubice, Czech Republic). 

ABTS method: The preparation of ABTS radical was adopted from the literature [34]. The working solution (3 mL) was mixed with elderberry extract (50 μL), and the decrease in absorbance was monitored at wavelength 734 nm after 10 min of reaction (in dark). Antioxidant activity was expressed as an equivalent amount of standard Trolox, using a calibration curve. The calibration solutions were prepared by diluting Trolox in methanol. The amount of standard Trolox added to the reaction agent was in the range of 0.003–0.069 μmol.

TPC determination: The working solution of the Folin–Ciocalteu reagent was prepared according to the literature [34], and 50 μL of diluted elderberry extracts were added. The results were expressed as milligrammes of gallic acid equivalents per gramme of elderberry. Calibration solutions were prepared by diluting gallic acid in water. The amount of gallic acid added to the reaction agent was in the range of 0.002–0.029 mg. 

TAC assessment: The preparation of buffer solutions was adopted from [35]. An amount of 200 μL of elderberry extract was added to 2.8 mL of buffers with pH 1 and pH 4.5, and their absorbance was recorded at wavelengths 520 and 700 nm. Each sample was measured five times. The wavelength of 520 nm corresponds to absorption maximum of cyanidin-3-glucoside, which is main anthocyanin present in elderberries. The wavelength was confirmed by the absorption spectrum of elderberry extract (Figure S1). The results were expressed as cyanidin-3-glucoside equivalent and calculated according to the formula below.

$$TAC\left(\mathrm{mg}/L\right)=\frac{{\left[(A\right.}_{520}\left.-A_{700}{)}_{\mathrm{pH}1}-{\left(A_{520}-A_{700}\right)}_{\mathrm{pH}4.5}\right]\cdot MW\cdot DF\cdot 1000}{\varepsilon \cdot l}$$

where *A* is the absorbance recorded at the given wavelength and buffer pH, *MW* is the molecular weight of cyanidin-3-glucoside (*MW* = 449.2 g/mol), *DF* is the dilution factor (0.2 mL of sample is diluted to 3 mL, *DF* = 15), *ε* is the molar absorption coefficient (26,900 L∙mol^−1^∙cm^−1^ for cyanidin-3-glucoside), and *l* is the cuvette pathlength (cm).

### 3.5. Statistical Evaluation of Experimental Data

Statistical analysis was performed in Statistica 12 (StatSoft CR, Prague, Czech Republic) and QC Expert 2.9 (Trilobyte, Pardubice, Czech Republic) at a significance level of 95% (α = 0.05). Calibration data were measured at eight concentration levels, each level five times (*n* = 5) or three times (*n* = 3) for spectrophotometric methods or HPLC analysis, respectively, and interpolated using linear least squares regression (QC Expert). Graphic diagnostics (Pregibon, Williams, and L-R graphs) together with Jackknife residuals were used to identify influential points. The significance of the regression intercept was tested using Student’s *t*-test, and the linearity of the calibration curves was verified by residual plots. The regression parameters together with their standard deviations and coefficients of determination are given in the Supplementary Materials (Table S1). The HPLC method was validated in terms of linearity, detection limits, precision, and repeatability. The coefficients of determination were greater than 0.999 for both standards, demonstrating high linearity. The instrumental limits of detection (LOD) and quantification (LOQ) were calculated as the concentration yielded signal-to-noise ratio of S/N = 3 and 10, respectively (Table S1). 

All experiments were repeated five times (*n* = 5) and three times (*n* = 3) for each sample for spectrophotometric methods and HPLC analysis, respectively. The results are presented as confidence intervals $\bar{x}$ ± *s.t*_1−_*_α_*, where $\bar{x}$ is the arithmetic mean, *s* is the standard deviation, and *t*_1−_*_α_* the critical value of Student’s t-distribution for five or three repetitions, 2.571 or 3.182, respectively. 

The discrimination of individual samples was performed using a principal component analysis (PCA) and factor analysis (FA) in Statistica 12. First, the normality of the data was verified by exploratory data analysis, and the Box–Cox transformation was applied to the data that did not show a normal distribution. The significance of the sample preparation was tested by one-way analysis of variance (ANOVA).

## 4. Conclusions

The four main anthocyanins present in elderberry fruit extracts were determined using reversed-phase HPLC with spectrophotometric detection. All anthocyanins were separated in 5 min on the Ascentis Express C18 column, using a rapid isocratic elution of aqueous methanol acidified with formic acid. Such fast separation of elderberry anthocyanins has not been presented so far. Furthermore, antioxidant capacity, total phenolic content, and total anthocyanin content were determined using spectrophotometric methods. All results obtained were evaluated by a principal component analysis.

The content of anthocyanins was found to be strongly dependent on the sample pretreatment. During drying, the anthocyanins degrade easily, and an extremely high decrease in the anthocyanin content was observed after the drying process. Therefore, freezing is definitely better for the storage of berries than drying them. The spectrophotometric results were consistent with those obtained by the HPLC method, which was proven by a high correlation coefficient. Therefore, these simple spectrometric methods, especial TAC, can be used for the rapid screening of elderberry samples on anthocyanin content. 

The principal component analysis has proven to be a useful tool for distinguishing the samples according to their pretreatment and has clearly divided the samples into two groups (drying vs. freezing). Furthermore, the effect of altitude on the discrimination of the samples was determined. The influence of other geographical parameters was not observed. The altitude is probably related to the ripeness of the berries and thus to the content of anthocyanins. 

## Figures and Tables

**Table 1:** The list of elderberry fruit samples analysed together with the place of harvest.

Sample	City	Region	Coordinates	Altitude	Pretreatment	Dry Matter (%)
1	Nové Město nad Metují	HK	50.331 N, 16.155 E	320 m	D, F	18.1
2	Nové Město nad Metují	HK	50.361 N, 16.149 E	320 m	D, F	21.6
3	Kutná Hora	CB	49.968 N, 15.287 E	260 m	D, F	19.0
4	Nové Město nad Metují	HK	50.336 N, 16.145 E	320 m	D, F	20.8
5	Blansko	SM	49.377 N, 16.569 E	400 m	D, F	15.3
6	Náchod	HK	50.406 N, 16.118 E	390 m	D, F	18.7
7	Náchod	HK	50.419 N, 16.199 E	390 m	D, F	14.6
8	Jaroměř	HK	50.351 N, 15.853 E	300 m	D, F	13.8
9	Hradec Králové	HK	50.217 N, 15.727 E	290 m	D, F	28.0
10	Chlumec nad Cidlinou	HK	50.150 N, 15.571 E	230 m	D, F	14.7
11	Nové Město nad Metují	HK	50.312 N, 16.134 E	300 m	D, F	22.0
12	Chlumec nad Cidlinou	HK	50.112 N, 15.484 E	220 m	D, F	21.4
13	Nové Město nad Metují	HK	50.330 N, 16.147 E	320 m	D, F	22.5
14	Nové Město nad Metují	HK	50.321 N, 16.124 E	320 m	D, F	17.8
15	Náchod	HK	50.417 N, 16.151 E	380 m	D, F	19.7

Abbreviations: HK—Hradec Králové, CB—Central Bohemia, SM—South Moravia, D—dried, F—frozen.

## Data Availability

The data presented in this study are available on request from the corresponding author.

## Supplementary Materials

The following supporting information can be downloaded at https://www.mdpi.com/article/10.3390/molecules28186690/s1. Figure S1: Spectra of elderberry extract; Figure S2: Significance of sample pretreatment; Table S1: Calibration parameters; Table S2: ANOVA results.
