# Supplementary material for: The Effect of Sample Pretreatment on the Anthocyanin Content in Czech Wild Elderberry (Sambucus nigra L.)
Source: Molecules. 2023 Sep 19;28(18):6690. doi: 10.3390/molecules28186690 (PMC10537702; doi:10.3390/molecules28186690)

Article

The Effect of Sample Pretreatment on the Anthocyanin Content in Czech Wild Elderberry (*Sambucus nigra* L.)

Lenka Česlová [1,*], Petra Kalendová [2], Lucie Dubnová [1], Marek Pernica [3] and Jan Fischer [1]

[1] Department of Analytical Chemistry, Faculty of Chemical Technology, University of Pardubice, Studentská 573, 53210 Pardubice, Czech Republic; luci-dubi@seznam.cz (L.D.); jan.fischer@upce.cz (J.F.)
[2] Department of Inorganic Technology, Faculty of Chemical Technology, University of Pardubice, Doubravice 41, 53210 Pardubice, Czech Republic; petra.kalendova@upce.cz
[3] Research Institute of Brewing and Malting, Mostecká 7, 61400 Brno, Czech Republic; pernica@beerresearch.cz
* Correspondence: lenka.ceslova@upce.cz; Tel.: +420-466037397

Keywords: Elderberry (*Sambucus nigra L.*); anthocyanins; HPLC, total anthocyanin content, total phenolic content, antioxidant capacity, principal component analysis

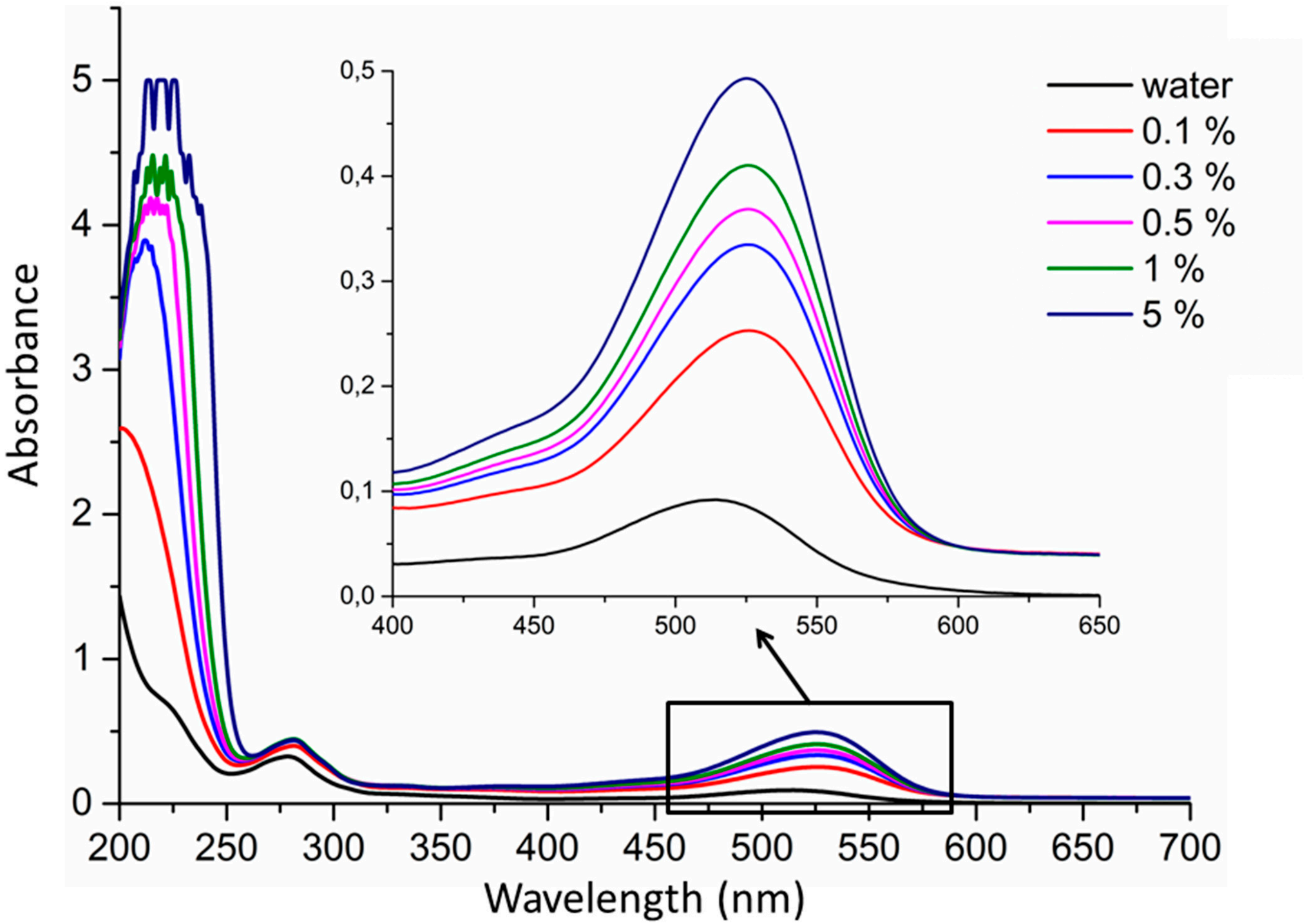

Figure S1. Spectra of elderberry extract. Wavelength range 200-700 nm, dependence of absorbance on amount of formic acid in solution (0-5% v/v).

Table S1. Calibration parameters.
Calibration ranges, regression parameters (with standard deviations), coefficients of determination (R^2), limits of detection (LODs), and limits of quantification (LOQs) for each standard used.

Standard	Concentration range	Calibration equation	R^2	LOD [mg/L]	LOQ [mg/L]
	**HPLC**				
Cyanidin-3,5-diglucoside	5 – 100 mg/L	y = 4.7 (1.4) x – 0.35 (0.03)	0.9996	0.10	0.33
Cyanidin-3-glucoside	5 – 100 mg/L	y = 13.1 (4.3) x + 1.1 (0.11)	0.9995	0.05	0.17
Cyanidin-3-sambubioside	5 – 100 mg/L	y = 12.4 (3.2) x + 1.0 (0.13)	0.9994	0.06	0.20
	**Spectrophotometric methods**				
Trolox	0.003 – 0.07 µmol	y = 1376 (6.1) x -1.5 (0.2)	0.9992	-	-
Gallic acid	0.001 – 0.020 mg	y = 45.2 (0.3) x + 0.019 (0.003)	0.9980	-	-

Table S2. ANOVA results.

Testing of sample pretreatment (frozen/dried) significance on obtained results. In case of HPLC the sum of anthocyanins content was used for calculation.

F = sample pretreatment

method	$F_{0.95}$ (4,25)	F_e	conclusion
HPLC	24.4	81.2	significant
TAC		65.7	significant
ABTS		45.8	significant
TPC		24.5	significant

Figure S2. Graphical representation of sample pretreatment significance. Weighted means were used. Vertical bars denote 0.95 confidence intervals.

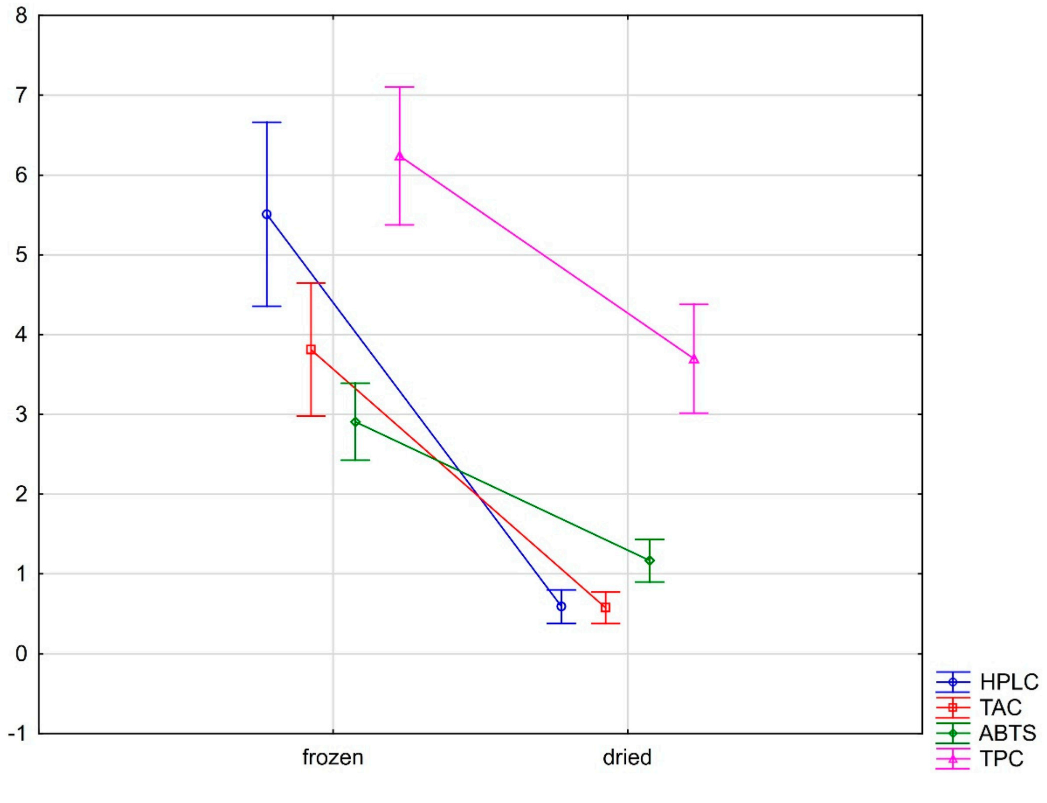

Supplement: Supplementary file 1 [file molecules-28-06690-s001.zip › molecules-2611335-supplementary.pdf]
